# Service user and caregiver involvement in mental health system strengthening in low- and middle-income countries: a cross-country qualitative study

**DOI:** 10.1017/S2045796017000634

**Published:** 2017-11-08

**Authors:** H. Lempp, S. Abayneh, D. Gurung, L. Kola, J. Abdulmalik, S. Evans-Lacko, M. Semrau, A. Alem, G. Thornicroft, C. Hanlon

**Affiliations:** 1 Faculty of Life Sciences and Medicine, Academic Rheumatology, King's College London, London, UK; 2 Department of Psychiatry, Addis Ababa University, College of Health Sciences, School of Medicine, Addis Ababa, Ethiopia; 3 Transcultural Psychosocial Organization (TPO) Nepal Baluwatar, Kathmandu, GPO Box 8974, Nepal; 4 Department of Psychiatry, College of Medicine, University of Ibadan, University College Hospital, PMB 5116, Nigeria; 5 Personal Social Services Research Unit, London School of Economics and Political Science Centre, London, UK; 6 Centre for Global Mental Health, Institute of Psychiatry, Psychology and Neuroscience, King's College London, London, UK

**Keywords:** Health system strengthening, low- and middle-income countries, mental health gap, mental health system, qualitative study, service user and caregiver involvement

## Abstract

**Aims.:**

The aims of this paper are to: (i) explore the experiences of involvement of mental health service users, their caregivers, mental health centre heads and policy makers in mental health system strengthening in three low- and middle-income countries (LMICs) (Ethiopia, Nepal and Nigeria); (ii) analyse the potential benefits and barriers of such involvement; and (iii) identify strategies required to achieve greater service user and caregiver participation.

**Methods.:**

A cross-country qualitative study was conducted, interviewing 83 stakeholders of mental health services.

**Results.:**

Our analysis showed that service user and caregiver involvement in the health system strengthening process was an alien concept for most participants. They reported very limited access to direct participation. Stigma and poverty were described as the main barriers for involvement. Several strategies were identified by participants to overcome existing hurdles to facilitate service user and caregiver involvement in the mental health system strengthening process, such as support to access treatment, mental health promotion and empowerment of service users. This study suggests that capacity building for service users, and strengthening of user groups would equip them to contribute meaningfully to policy development from informed perspectives.

**Conclusion.:**

Involvement of service users and their caregivers in mental health decision-making is still in its infancy in LMICs. Effective strategies are required to overcome existing barriers, for example making funding more widely available for Ph.D. studies in participatory research with service users and caregivers to develop, implement and evaluate approaches to involvement that are locally and culturally acceptable in LMICs.

## Introduction

For many years, people with severe mental illness have been considered to be chronically disabled, unable to make responsible decisions, and limited in their capacity to engage in productive activities. Such negative views have also contributed to the social exclusion and stigmatisation that are commonly experienced (Chamberlin, 2005; Corrigan *et al.* 2012; Mizock *et al.* 2014). The introduction of new care models in health, e.g. the person-centred approach, the recovery model, and shared-decision-making, has led service users and their caregivers to take a greater role in shaping the service they receive (Wallcraft *et al.* 2011; Storm & Edwards, 2013). The WHO has promoted service user and caregiver involvement in health care (World Health Organization, 2001, 2010, 2013).

There is a growing emphasis on the value of participatory approaches at the level of health care systems (Crawford *et al.* 2002; Chamberlin, 2005; Thornicroft & Tansella, 2005; Tambuyzer *et al.* 2014; Wallcraft *et al.* 2011). Involvement of individuals with personal experience of mental illness and their caregivers in mental health system activities (policy-making, planning, service delivery, quality improvement, monitoring, service evaluation, research, education and advocacy) has increased in recent years in many countries (Nilsen *et al.* 2006; Tambuyzer *et al.* 2014; Wallcraft *et al.* 2011; Newman *et al.* 2015). Such service user and caregiver involvement can take place at individual or *micro-level* (e.g. in individual care planning, assessment and care management); at health organisation/community level or *meso-level* (e.g. in local service planning, monitoring and evaluation, advocacy, training and recruitment of staff, input into guidelines); or at a strategic level or *macro-level* (e.g., policy-making, national level planning and advocacy) within the mental health system (Hickey & Kipping, 1998; Tritter & McCallum, 2006; Tambuyzer *et al.* 2014).

There is a growing consensus on the positive value of service user participation in the mental health system (Crawford *et al.* 2002; Tait & Lester, 2005; Whiston *et al.* 2017). Such participation can lead to more accessible and acceptable health services, enhance service development and improve the responsiveness of mental health services (Crawford *et al.* 2002; Goodwin & Happell, 2008; Tambuyzer *et al.* 2014; Bee *et al.* 2015). Additional reported benefits include better therapeutic relationships, improved self-esteem and confidence, greater empowerment in relation to recovery and increased service satisfaction (Crawford *et al.* 2002; Thornicroft & Tansella, 2005; Henderson *et al.* 2009; Tambuyzer *et al.* 2014; Omeni *et al.* 2014; Whiston *et al.* 2017), compared with traditional models of care.

In low- and middle-income countries (LMICs), mental, neurological and substance use (MNS) disorders contribute significantly to the burden of disease (Prince *et al.* 2007; Thornicroft *et al.* 2010). Yet they are characterised by huge treatment gaps (Kohn *et al.* 2004) and the health systems often fail to meet the needs of people with MNS (Semrau *et al.* 2015). Service user and caregiver involvement has been proposed as an essential means of strengthening weak mental health care systems (Saraceno *et al.* 2007; Wallcraft *et al.* 2011) and of improving mental health quality of care (Hanlon *et al.* 2010; Thornicroft *et al.* 2010; Raja *et al.* 2012). Yet at present it is still the case that service users are often denied the right to health, to full citizenship and meaningful participation in clinical decision-making (Kleintjes *et al.* 2013; Semrau *et al.* 2016).

This study was conducted as part of the multi-country Emerging mental health systems in LMICs (Emerald) programme, which investigates the health system requirements for successful scale-up of integrated mental health care in six LMICs (Ethiopia, India, Nepal, Nigeria, South Africa and Uganda) (Semrau *et al.* 2015). Recent situational analyses have been published from Emerald project countries describing the system and clinical level characteristics and resources for integrating mental health care into primary care in the study sites (Semrau *et al*. 2015; Abayneh *et al.* 2017; Mugisha *et al.* 2017), while service users’ and caregivers’ involvement in mental health strengthening are reported by Semrau *et al.* in this edition, and in the web appendices to this paper.

Against this background, the aims of this paper are to: (i) explore the *experience* of involvement of mental health service users and their caregivers in mental health system strengthening; (ii) analyse the potential *benefits and barriers* of such involvement; and (iii) identify strategies required to achieve greater service user and caregiver participation.

## Method

The study design was a cross-sectional, using qualitative individual in-depth interviews with service users, caregivers, policy makers and heads of mental health services in Ethiopia, Nepal and Nigeria. The sample selection method was purposive sampling, based on the close knowledge of researchers of the local community in each site. A common interview guide was developed and adapted to the local context by the in-country research teams. All provided informed consent prior to the interviews. For those who were unable to read or write, an independent witness confirmed that the information had been explained as per the Participant Information Sheet and a thumb print was sought from the individual. The average interview duration was 40 min. A pilot study was conducted in Nepal and Nigeria with two and three patients, respectively. Following the pilot study, minor amendments to the interview guide content were incorporated, to reflect the feedback of interviewees and research teams. Interviews were audio-recorded and transcribed before data analysis. The content of the interview topic guide addressed questions related to: existing level of involvement and its potential benefit in mental health policy-making, mental health planning and service development, monitoring the quality of mental health services and service evaluation.

### Data analysis

A thematic approach was employed for the data analysis (Braun & Clarke, 2006). A cross-country analysis (three sites) was conducted from all 83 individual interviews to identify commonalities and differences, assisted with Open Code 4.02 (Umeå University, 2013) or NVivo 10, qualitative software computer programmes (Seale, 1999*a*). Interviews were transcribed verbatim in the respective languages. All the interviews were then translated into English by other research team members; selected audio files and transcripts were cross-checked for accuracy before coding. After thorough familiarisation with the data through repeatedly listening to the audio files and reading through the transcripts, team members coded transcribed interviews independently, compared the codes and reached consensus if there was a disagreement. The key themes and sub-themes were derived from the primary codes following further cross-checking by team members. A comparative analysis was completed between the categories of respondents and illustrative accounts were identified. The results were then interpreted in relation to the conceptual approaches of Tambuyzer *et al*. (2014) and Carman *et al*. (2013).

### Validation of qualitative data

The validation of the study data was undertaken by cross-checking the emerging themes during the data analysis against the transcript data with: (i) all the field researchers who had gathered the qualitative data; (ii) service users/caregivers in each site; (iii) mental health advocates, and by; (iv) the inclusion of deviant cases (Seale, 1999*b*). Moreover, initial codes from the pilot study and from the first interviews were discussed with the research team in each country and any divergent codes were identified and an agreement was sought for inclusion or exclusion of the codes (Barbour, 2001; Malterud, 2001).

## Results

The number and nature of the participants are shown in Table 1. None of those invited to participate refused to contribute (Barbour, 2001; Guest *et al.* 2006). The qualitative data from the cross-country study resulted in an analytical framework that consisted of four key themes, within health care system domains where the experiences of involvement by participants were reported and operationalised, as summarised in Table 2. The four key emergent themes identified were: (i) experience of servicer user/caregiver involvement; (ii) barriers to involvement; (iii) potential benefits of involvement; and (iv) strategies for greater involvement.

**Table 1.** Process of data gathering across three LMICs included in the Emerald Programme

Country	Ethiopia	Nepal	Nigeria
Year of data collection	2014/15	2014	2013/2014
Trained and experienced researchers	Four (two male/two female)	Six (two males/four females)	One (female)
Location of 1:1 interviews	Mental Health Research Office, private facilities	Service User organization offices, homes, health facilities	Homes, University College Hospital premises
Language: translated into English by bilingual researchers	Amharic	Nepali	Yoruba
Recruitment sample type	Purposive (gender, age, level of education, religion, duration of mental health service use of service users/caregivers*)	Purposive (gender, role, duration of mental health service use of service users/caregivers^†^)	Purposive, gender, age duration of mental health service use
Number of participants and roles	39 Service users (*n* = 13) with severe mental disorders (schizophrenia, schizoaffective disorder, bipolar disorder or major depressive disorder with psychotic features), caregivers (*n* = 10), heads of primary care facilities (*n* = 8) and policy makers/planners/services developers (*n* = 8)	24 User advocates and representative of Service Users organisations (*n* = 7); caregiver affiliated to organisation (*n* = 1); Service Users not affiliated to any organisation (*n* = 7); Caregivers not affiliated to any organisations (*n* = 9)	20 Service users (*n* = 10); Caregivers (=10) from a support group affiliated to the Department of Psychiatry

* For details see Abayneh *et al.* (2017).

† Gurung *et al.* (2017).

**Table 2.** Four main themes and subthemes of experiences, barriers, potential benefits and strategies for service users/caregiver involvement across the three LMICs

Themes and subthemes	Ethiopia	Nigeria	Nepal
1) *Experiences of Involvement*			
1a) No or limited experiences of involvement*†‡	X	X	X
2b) positive experience of involvement outreach)*†‡	X	X	
2) *Barriers to involvement*			
2a) Poor access to mental health service*†‡	X	X	X
2b) Multiple level problem of stigma (self-stigma, community, service provider and within the health system)*†‡	X	X	X
2c) Power differentials in health system (lack of: personal confidence, knowledge of services, support in local community)^†^	X	X	X
3) *Potential benefits of involvement*			
3a) Sharing lived realities and experience to improve lives of service users/care givers*†	X	X	X
3b) Contributions to improvement of mental health services quality^†^	X		X
3c) Awareness raising and service promotion^†^	X	X	X
4) *Strategies for greater involvement*			
4a) Service users and caregiver mobilisation and empowerment†‡	X	X	X
4b) Training service users/caregiver and service providers†‡	X	X	X
4c) Ensure human rights for greater involvement*†‡	X	X	

* *Macro-level* (e.g. policy-making, national level planning and advocacy).

† *Meso-level* (e.g. in local service planning, monitoring and evaluation, advocacy, training and recruitment of staff, input into guidelines).

‡ *Micro-level* (e.g. individual care planning, assessment and care management).

In the following sections, under each of the four main themes illustrations of 2–3 subthemes are presented (coded accounts supporting the sub-themes can be obtained from co-authors SA, DG LK).

### Theme 1. Experience of servicer user/caregiver involvement

For many service users and caregivers, involvement in systems processes appeared to be a somewhat alien concept as they had little direct experience.

#### No or limited experiences of service user involvement

“After I have been sick I have never been involved, whether in policy making or in a meeting… because I am sick, nobody is accepting what I am saying, people used to say I have no proper expression…If you are a sick person… uhh…I had been unemployed for a long time, even after I got better…”. (Service user, Ethiopia)

One participant reported a low level of involvement due to lack of interactions with people outside her home.

“I have never been outside my home [not interacted much outside home]. How can a person like that know these things [how to improve mental health services]”? (Service user, female, Nepal)

A minority stated that other health issues were discussed in user groups, rather than focusing on mental health service improvement.

“In the user association which I belong [to], we meet regularly to encourage ourselves and learn more about staying healthy that is all we do. We do not discuss involvement with service provision. We leave that to those taking care of us [service providers]”. (Service user, male, Nigeria)

#### Positive experience of service user involvement

Although most participants lacked experiences of involvement, a few reported limited opportunities to voice their opinions about service improvement, or drew examples from non-mental health services, as in Ethiopia and Nigeria.

“For example, previously the nurses were so ignorant, they even don't consider the pregnant woman who is about to deliver, but now there are a lot of improvements, with many meetings and discussions… Today, no professional in the hospital or health centre will mistreat a pregnant woman… because we told them [staff]. And just like that I believe if we do the same for the mental health, we can bring change, if we discuss with the relevant people… ”. (Caregiver, Ethiopia)

One service user contributed positively by expressing his personal view about involvement and the potential of non-medicinal treatment.

“I was invited to a meeting in Abuja [capital] to discuss my views on how to improve mental health services in hospitals. I gave my views on the need for those taking care of people with mental illness to involve them [users] more in treatment and not just give them medications without options. It was a positive experience”. (Service user, male, Nigeria).

### Theme 2. Barriers to involvement

The interviewees mentioned a range of obstacles they encountered when living with MNS conditions in Ethiopia, Nepal and Nigeria, such as logistical, economic and attitudinal barriers.

#### Difficulties to access treatment and care

Many service users and caregivers voiced their concerns about access to treatment and care, in countries where mental health services are poorly resourced and where public transport is non-existent or unaffordable.

“…Yes, there is a problem. We live far away from this hospital, so we are expected to travel longer distance to get the service. We get the medicine every two or one month. I bring my daughter may be once or twice a year for a check-up”. (Caregiver, Ethiopia)

“What can we do? If there is a need to go to Kathmandu then there is no money. Even while going to Narayanghat [local town], it takes hundred rupees and there is no money to spare”. (Caregiver, male, Nepal)

Lack of employment opportunities, and therefore the absence of a regular income, are for many participants a major difficulty.

“Finances can be a major barrier, as most patients are not employed and so may not have the money for their treatment and drugs. The government also needs to increase priority attention and funding for mental health. This will reduce stigma and improve awareness”. (Caregiver, male, Nigeria)

#### Stigma and discrimination

Recipients and care providers reported the impact that the fear of stigma and discrimination had on them.

“… when sometimes I discuss this [service user involvement] with some colleagues who are sometimes very experienced psychiatrists, … I have the feeling that they're very sceptical about this and that the overall mentality is that what people with mental disorders think doesn't really matter to what we do. So that is not really the best approach to promote such initiatives…. and I'm afraid… lay persons share the same mentality as policy makers. (Policy-maker/planner, Ethiopia)”

Others voiced their concerns about how long-standing engrained attitudes by officials contributed to an observed reluctance to invite service users to participate actively.

“When there is discrimination the service users won't be selected for the participation. They [policy makers] might think why we should not involve these mad [people] for making policy. So far in our politics, politicians are cunning, this has been a tradition since a long time. The policy makers won't allow service users to make policy”. (Service user, female, Nepal).

#### Power differentials between recipients and service providers

Service users and caregivers in all three LMICs painted a particularly critical picture of the lack of professionalism of health care staff towards patients who attend the mental health services, with little opportunity for redress.

“There must be some ego issues regarding the power. Or they [health professionals] don't want to change their orthodox view, thinking we can't do anything. The people in power are driven by objectives, they don't see the patient as a person”. (Service user, female, Nepal)

Some service users and caregivers expressed lacking knowledge and confidence to participate effectively in mental health system strengthening, due to lack of education.

“Well, most of the time people with mental health problem are shy and fearful. They have low self-esteem so they don't approach the professionals to express their ideas and opinions”. (Caregiver, Ethiopia

“… We (caregivers) need to know what we have to do, at all levels, so that we will have acceptance by the people whom we are going to work together. We need to know the regulations, rules related to our responsibilities… we must know the extent and the limits of our rights and what duties we have to carry out”. (Caregiver, Ethiopia)

Some talked about how their lack of education was part of the reasons why they were reluctant to share their experiences.

“Uneducated people like us won't be able to say much [mental health system meetings). The educated people may be able to though. I don't think that uneducated people like us can really contribute.” (Service user, female, Nepal).

Finally, the perceived lack of support in local communities by policy makers was highlighted as a disappointment and hindered facilitation of active involvement in mental health affairs.

“…I do believe, definitely, it is also our [policy makers] responsibility to help them [service users/caregivers] organize, but we are not doing a good job. We are not doing anything actually…. Well, in other words, the families and the user groups are not sophisticated and it is not encouraged… it takes some resources to organize for this group….they need to meet, they need paper, and they need a little bit of an office… it is just not easy… and also the stigma…”. (Policy-maker/planner Ethiopia)

### Theme 3. potential benefits of service user/care giver involvement

Interviewees identified three important aspects that gave them confidence, if given the opportunity by mental health service managers and policy makers, to contribute positively to mental health service strengthening that can benefit individuals, families and communities.

#### Lived realities and experiences

Participants expressed how their ‘lived experiences’ of their mental health condition can be an important contribution to improve the lives of patients with long-term psychiatric illness, including the caregivers who look after family members, when taken seriously and being listened to.

“…. we (service users) have to go and discuss with them (service providers) and we have obligation to go and talk to them because we are the victims. … so, as long as we are the victims and the people suffering from the problems we should go and tell the professional how we are suffering from the medications, that is our obligation….uhh …”. (Service user, Ethiopia)

Caregivers’ provide round-the-clock attention for family members with mental illness, and can share their experiences with other stakeholders that can be utilised for mental health system strengthening.

“We know more about the problem of service users as they are the ones looking after them 24 h a day. This helps to bring insider's perspective in the process”. (Caregiver, female, Nepal)

“A sufferer or someone that has recovered from mental illness is in the best position to contribute to decision-making with regards to care of those with the illness. In fact, users should be the major contributors in decisions taken by the hospital and government towards our care, because we are the ones that will use the services”. (Service user, male, Nigeria)

#### Contributions to I improvement of local mental health service quality

Interviewees expressed how their direct contributions, have also an important role to play in raising the standard and quality of mental health service delivery, including research.

“Well, I think it [involvement] will be important because it will help people with mental health problems to have control about the quality of services they receive and manoeuvre the way their problem is addressed. It [involvement] can also help protect people with mental health problem from any abuse and maltreatment. Their [service users and caregiver] participation could also mean that the professionals can get needed information from them about their need and situation”. (Service user, Ethiopia)

The plea for participation of all stakeholders was also voiced by several interviewees.

“The health system should be inclusive. While making the [mental health policy], emphasis should be given to the inclusive health system”. (Service user, male, Nepal)

Partnership in research was highlighted as a strength in providing relevant information.

“When you involve patients in your research, the results must be very good, because we have all the information you need and we have direct experience of these conditions”. (Service user, female, Nigeria)

#### Awareness raising and service promotion

Participants explained that experiencing well-functioning and caring mental health services can inform people in the community about how mental illness can be treated and may lead to a positive image in the local community.

“I think they [service users] can contribute more than anyone; first if they got better because of the treatment …. if that person gets better, people in that surrounding [around that person] will know/be aware of what to do when they see other people with this problem [mental illness]…”. (Policy-maker/planner, Ethiopia)

“If patients are involved in monitoring services, it will provide credible information that will boost the quality of services. It will also encourage other patients [to attend mental health services].” (Caregiver, male, Nigeria).

### Theme 4. Strategies for greater service user and caregiver involvement in mental health strengthening

Service users and caregivers in all three countries were forthcoming with ideas on how best to raise awareness about the role of service users and their families to improve mental health services.

#### Service user and caregiver mobilisation and empowerment

Working together with the media was given as an example of how people's attitudes, behaviour and knowledge can be changed towards people with mental illness and their families.

“You can get help from the media. Certain messages can be relayed through the mainstream media and feedback can be gathered…. You can also organize a documentary like dramas by taking characters of 4–5 people and writing script inspired by the real life events”. (Service user, male, Nepal)

Others suggested, in collaboration with service providers, to further the progress of mental health service provision by generating a force for change (a lobby group) that results in being taken more seriously by policy makers.

“We need to make service users understand about what happens if there is a good [mental health] policy, what is a policy, what happens in policy etc. We need to empower them [service users/caregiver] we need to make them understand that the policy environment would be good”. (Service User, male, Nepal)

#### Need for training for recipients and providers of mental health care

The need for comprehensive training for mental health care staff, families and communities on a range of aspects of mental health was voiced by many interviewees with the aim not solely focused on treatment and care, but also about social issues, such as abuse by staff, stigma interventions, and respect for human rights.

“Here everybody should know his right and responsibility [training for service users/ cares]…if stakeholders do their work we can improve [the] quality of [the] service. First of all: community system, sociology type of trainings to understand the society better, because we only know heath related things… as well as short term trainings on specific [mental] health matters.”. (Health Center Head, Ethiopia)

Some suggested that training on how to overcome stigma may lead to the greater disclosure of community members talking about their mental illness.

“The awareness training is needed whether it is done by the NGO [non-governmental organization] or government. They [service users] should be given their rights and the social stigma, which they fear, should be removed. They [service users] shouldn't be treated like the second types of the citizen. They [service users] will come into the open if they are treated like the normal person”. (Service user, male, Nepal)

Others observed how education and public awareness campaigns in other long-term conditions, such as HIV/AIDS have contributed to positive attitudinal, behavioural and knowledge changes in local communities, when funding was made available.

“Service users need to know more about mental illness and its care, before we face others to talk confidently about mental illness. Many service users currently don't even know what to ask for. If you look at what is happening in the areas of HIV/AIDS, there are lots of programs by different groups that sufferers are engaged in, that educate them about the illness. We also need such support and encouragement and training”. (Service user, female, Nigeria)

#### Respect for human rights for recipients of mental health services

Finally, service users in Ethiopia and Nepal raised the important issue of human rights that is deeply linked to mental health service provision in many countries, but did not seem to receive the same degree of respect, according to some interviewees, as one participant explained:

“Nowadays there is a right-based approach to mental health. But apart from the legal capacity for the service user, the main issue is … that they are not eligible [due to mental illness], to be involved in many activities…. although they [service users/caregivers] have lots of experiences, they are unable to take their decisions. Just because I am in the delusion or the hallucination doesn't mean someone should come up and take decisions for me.” (Service user, female, Nepal)

## Discussion

Our analysis showed that service user and caregiver involvement in the health system strengthening process may have been seen as a rather foreign concept for most participants. They reported very limited access to direct participation. In the few cases where service users did participate in this way, their experience of involvement was mostly poor and entailed limited ‘tokenistic’ engagement. In Nigeria a few service users reported positive experiences of involvement in health service improvement, which they hoped could benefit other service user groups. Such encouraging experiences suggest that they viewed their invitations as a step forward towards future involvement in decisions that affect their care. At the same time, however, some (indeed a minority in Ethiopia) service users and carers had very practical ideas of how they could become involved in planning and service provision issues, as presented in the sub-theme ‘lived realties and experiences’.

A similar trend was reported in the areas of involvement across all three countries for example in varying responses that described the largely very limited experience of service users and caregivers of engagement. Although historically theories about service users and caregivers had been generated through research ‘on them’, recent trends have shifted towards collaborative or user-led research where service users and caregivers actively generate research questions and conduct (action) research, especially in high-income countries (Munn-Giddings & Winter, 2013).

Several common barriers across all three countries were reported as reasons for the dearth of any or meaningful involvement. Stigma and poverty were highlighted by participants as the major barriers. Mental health stigma in the communities and within health systems is prevalent across countries and cultures that hinders the mental health system strengthening process and impedes recovery of mental illness (Saraceno *et al.* 2007; Evans-Lacko *et al.* 2012; Clement *et al.* 2015; Lewer *et al.* 2015). This study shows that stigma is also the major barrier to service user and caregiver participation. Feelings of disempowerment are commonly reported among service users as a result of stigma (Corrigan *et al.* 2014). This was emphasised by participants from Ethiopia and Nepal who mentioned not having the know-how or feeling empowered enough to participate and interact in health system processes. Structural stigma (Corrigan *et al.* 2004) resulting in low prioritisation of mental health care in public health systems and discriminatory attitudes and behaviours of health workers were also listed as barriers restricting service users/caregivers to contribute to any decision-making and system-related processes. This was especially apparent in Ethiopia and Nepal, where service users were viewed as passive patients/caregivers by the health workers rather than active decision makers. A study conducted in Nepal on mental health system governance reported strong opposition of some national level policy makers who viewed service user involvement in policy-making as irrelevant (Upadhaya *et al.* 2017).

Poverty has been identified as a major barrier for addressing the treatment gap for mental health care in LMICs (Saxena *et al.* 2007) and is shown to be both the cause (social causation) and outcome (social drift) of mental illness (Lund *et al.* 2011). Most participants across our study sites cited poverty and lack of economic resources as a major barrier to treatment as well as to their involvement in systems level processes. They stated that due to low resources, their priority was to fulfil their basic human needs (food, accommodation, income, education) rather than seeking treatment or being involved in systems level processes.

Several strategies have been identified in our study that facilitates service user and caregiver involvement in the mental health system strengthening process. Support to access treatment, mental health promotion, and empowerment of service users was identified as necessary to overcome some of the existing barriers. This study suggests that capacity building for service users, and strengthening of user groups would equip them to contribute meaningfully to policy development from informed perspectives. McKinlay & Yiannoullou (2011) identified the importance of knowledge and skills in ensuring active and meaningful participation by recipients of care in mental health care policy development. The need for collaboration among service user groups to increase the power of advocacy through the ‘strength of numbers’ was emphasised. Group efforts will increase the chances of effective advocacy and have their voices heard in the community and their demands met by decision makers. In view of the relative neglect of mental health on the global health agenda and the limited support for representative organisations, we recommend an increase in funding to escalate service user representation.

In relation to a conceptually based interpretation of the findings, we have drawn upon on two conceptual frameworks: (i) Tambuyzer *et al.* 2014 who have outlined the processes of patient involvement, e.g. this model provides an overview of the defining elements of patient involvement (e.g. power dimensions, organisational levels) and its determinants (e.g. attitudes, resources) and short-term outcomes (e.g. quality of care, empowerment); and (ii) the multi-dimensional model that considers patient engagement across the health care system, from the direct care setting to incorporating patient engagement into organisational design, governance and policy-making (Carman *et al.* 2013). In our view, our analysis is in line with the Tambuyzer *et al.* (2014) conceptual model because we address the conceptualisation of involvement, the barriers and facilitators (determinants of involvement), the benefits of involvement and strategies for greater involvement. Our analysis, however, is also aligned with the Carman *et al.* (2013) conceptual framework that identifies multidimensional elements of patient and family engagement, especially in relation to the micro, meso and macro levels of policy-making (see Table 2).

This study has several limitations. First, it presents views of service users/caregivers, head of health centres and policy makers from three countries and therefore provides a broad and in-depth perspective of their involvement in mental health care in LMICs. All three country research teams agreed on a common approach to the study and were able to incorporate local/cultural adaptations, e.g. content of topic guide, identification of different service user/caregiver commitments. The results however might not be generalisable due to the qualitative study design and the recruitment that was based on a purposive sample. Further, the purposive nature of the sampling means that this is a weaker methodology than other sampling approaches, such as random sampling. A somehow unusual aspect of the study, however, is that all the participants who were invited to be interviewed agreed to do so, which is rather atypical for this type of study.

## Conclusion

Involvement of service users and their caregivers in mental health decision-making is still in its infancy in LMICs. Effective strategies are needed to overcome existing barriers to such participation, for example making more widely available funding Ph.D. studies for participatory research with service users and caregivers to develop, implement and evaluate approaches to involvement that are locally and culturally acceptable in LMICs, which are already in place through the Emerald programme in Ethiopia and India.
